# Hydrogen sulfide-induced post-translational modification as a potential drug target

**DOI:** 10.1016/j.gendis.2022.03.022

**Published:** 2022-04-20

**Authors:** Hao-Jie Chen, Lei Qian, Ke Li, Yang-Zhe Qin, Jing-Jing Zhou, Xin-Ying Ji, Dong-Dong Wu

**Affiliations:** a School of Basic Medical Sciences, Henan University, Kaifeng, Henan 475004, China; b Henan International Joint Laboratory for Nuclear Protein Regulation, Henan University, Kaifeng, Henan 475004, China; c Kaifeng Key Laboratory of Infection and Biological Safety, School of Basic Medical Sciences, Henan University, Kaifeng, Henan 475004, China; d School of Stomatology, Henan University, Kaifeng, Henan 475004, China

**Keywords:** Hydrogen sulfide, Modification, Phosphorylation, S-nitrosylation, S-sulfhydration

## Abstract

Hydrogen sulfide (H_2_S) is one of the three known gas signal transducers, and since its potential physiological role was reported, the literature on H_2_S has been increasing. H_2_S is involved in processes such as vasodilation, neurotransmission, angiogenesis, inflammation, and the prevention of ischemia-reperfusion injury, and its mechanism remains to be further studied. At present, the role of post-translational processing of proteins has been considered as a possible mechanism for the involvement of H_2_S in a variety of physiological processes. Current studies have shown that H_2_S is involved in S-sulfhydration, phosphorylation, and S-nitrosylation of proteins, etc. This paper focuses on the effects of protein modification involving H_2_S on physiological and pathological processes, looking forward to providing guidance for subsequent research.

## Introduction

Hydrogen sulfide (H_2_S) is the third gas signaling molecule synthesized in mammalian cells after carbon monoxide and nitric oxide. In the past 30 years, the physiological and pathological processes of H_2_S in the human body have been studied extensively.^1^ H_2_S has long been considered a toxic gas with a rotten egg odor, but it has now been shown to be an important gas signaling molecule in both prokaryotes and eukaryotes.^2^^,^^3^ H_2_S is widely present in mammals and acts as a signaling molecule in the central and peripheral nervous system, cardiovascular, immune, endocrine, reproductive, and digestive systems,^4^, ^5^, ^6^, ^7^, ^8^ mediating diastolic and vasculogenesis, cell death, inflammation, and anticancer or carcinogenic effects.^9^, ^10^, ^11^, ^12^, ^13^

Post-translational modification (PTM) refers to the chemical modification of proteins after translation. For most proteins, this is a later step in protein biosynthesis. Precursor proteins are inactive and often undergo a series of post-translational processes before they become functional mature proteins. The types of processing are varied and generally fall into the following categories: phosphorylation, methylation, acetylation, sumoylation, and ubiquitylation.^14^, ^15^, ^16^, ^17^, ^18^ When proteins are made, 20 different amino acids are combined to make them. After protein translation, other biochemical functional groups of the protein (e.g., acetate, phosphate, different lipids, and carbohydrates) can attach to the protein to alter its chemistry, or cause structural changes (e.g., the establishment of disulfide bonds) to broaden its function.^17^

This paper aims to explore the PTM process in which H_2_S participates and its role in organisms, hoping to inspire researchers.

## Anabolism of H_2_S in the body

### Enzymes that synthesize H_2_S

H_2_S synthesis *in vivo* is affected by a variety of enzymes, three of which are best understood: cystathionine β-synthase (CBS), cystathionine gamma-lyase (CSE), and 3-mercaptopyruvate sulfurtransferase (3-MST).^19^ Naturally, CBS and CSE are mainly located in the cytoplasm,^20^ while 3-MST is mainly present in mitochondria and cytoplasm.^21^^,^^22^ However, under the condition of hypoxia, the mitochondrial-targeted sequence at the c-terminal of CBS can be recognized by Hsp70 and transported to the mitochondria, while CSE enters the mitochondrial cavity mediated by Translocase of the Outer Membrane 20^20^^,^^23^. The optimal pH of CBS and CSE was 8.5–9.0, and that of 3-MST was 7.4,^24^, ^25^, ^26^ but the pH of cytosol was 7–7.4, and the pH of the mitochondrial cavity was 8.0, which was weakly alkaline.^27^ Under hypoxic conditions, CBS and CSE are transported to the mitochondrial cavity, which is favorable for H_2_S production under this pH condition, this may be an autoregulation mechanism of cells under hypoxia. The substrates catalyzed by these three enzymes are l-cysteine, l-homocysteine, and 3-mercaptopyruvate, and then little amounts of l-cystine and d-cysteine. Among them, l-cysteine and l-homocysteine are the main substrates of CBS and CSE, its catalytic reaction requires the participation of cofactor pyridoxal-5-phosphate,^28^ 3-mercaptopyruvate is the important substrate of cysteine aminotransferase: 3-MST Axis.^29^^,^^30^ Meanwhile, d-amino acid oxidase in mitochondria and peroxisome catalyzed H_2_S formation from d-cysteine with the assistance of 3-MST,^31^^,^^32^ while selenium-binding protein 1, recently discovered in adipocytes, catalyzes the production of H_2_S, formaldehyde, and H_2_O_2_ from methanethiol.^33^ The specific reactions catalyzed by these enzymes have been discussed in several review.^34^^,^^35^ But these enzymes are expressed differently in different tissues (cells) and produce H_2_S with different efficiency, how do these enzymes work together to regulate the production/distribution of H_2_S is unclear.

### H_2_S metabolism

H_2_S is discharged from the body mainly by excretion, and respiration accounts for only a very small part of the excretion.^36^ Before excretion, H_2_S needs to undergo enzyme-induced catabolism and non-enzyme-induced catabolism, and enzyme-induced catabolism is the main pathway.^35^ In the mitochondria, H_2_S is catabolized by the sulfide quinone oxidoreductase system (SQR) to form SQR-bound cysteine persulfides, which are then broken down into thiosulphates (a reversible process) and also into sulfites and sulfates, which are excreted in urine.^37^^,^^38^ H_2_S can also be decomposed into sulfites by ethylmalonic encephalopathy 1 in the mitochondrial matrix.^39^ In the non-enzymatic way, the spleen excretes the sulfhemoglobin formed by the combination of H_2_S in red blood cells with methemoglobin.^40^

**Figure 1:**
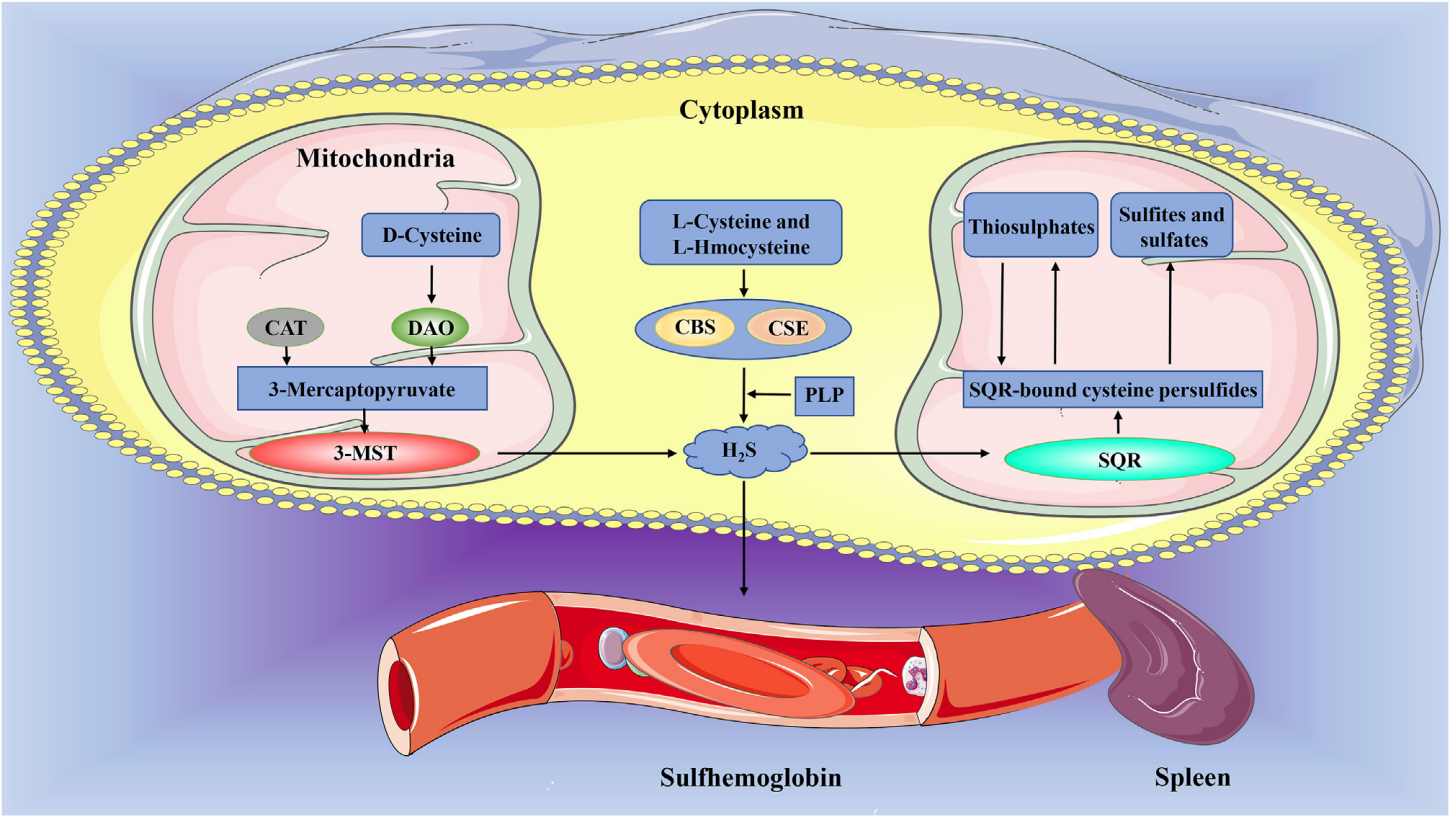
The main way of synthesis and metabolism of H_2_S. Endogenous H_2_S is mainly produced by three enzyme-catalyzed substrates of CBS, CSE, and 3-MST, and its path mainly includes enzymatic and non-enzymatic degradation. CAT: cysteine aminotransferase; CBS: cystathionine β-synthase; CSE: cystathionine gamma-lyase; DAO: d-amino acid oxidase; 3-MST: 3-mercaptopyruvate sulfurtransferase; PLP: pyridoxal-5-phosphate; SQR: sulfide quinone oxidoreductase system.

The rate of H_2_S production in living organisms is very fast, such as in the liver, kidney, and brain of mice, but high concentrations of H_2_S are harmful to mammals, and the level of H_2_S in tissues must be strictly controlled, suggesting that the production and metabolism of H_2_S is a tightly regulated and relatively balanced process^41^, ^42^, ^43^ (Fig. 1).

## H_2_S and S-sulfhydration

S-sulfhydration, also known as S-sulfuration or S-persulfidation, is the recently discovered H_2_S or polysulfide-induced PTM, which forms persulfides by chemically modifying specific cysteine residues of the target protein.^44^ Polysulfides can transfer sulfur atoms to cysteine residues with low pKa,^45^ convert cysteine –SH groups to –SSH, leading to protein S-sulfhydration and cell signal transduction.^46^^,^^47^ There is growing evidence showing that this PTM contributes to the signal transduction mediated by H_2_S in mammals and participates in the physiological and pathological processes of the cardiovascular system, urinary system, nervous system, and reproductive system (Table 1).

**Table 1:** Sites and functions of protein S-sulfhydration in different systems.

Location	Protein	Site(s)	Functions	Refs.
Cardiovascular system	PPARγ	Cys139	Adipogenesis	^53^
	PTP1B	Cys215	Restore ER stress homeostasis	^56^
	MuRF1	Cys44	Protect cardiac structural protein	^59^
	Keap-1	Cys151	Anti-oxidative stress	^60^^,^^61^
	PDI	Cys53/Cys57 and Cys397/Cys400	Reduce ER stress	^65^
	HuR	Cys13	Preserve endothelial cell function and delay atherogenesis	^66^
	TRPV4	Unknown	Vasodilatation	^71^
	TRPV1	Unknown	Vasodilatation	^72^
	CaMKII	Cys6	Improve mitochondrial function	^75^
	MEK1	Cys341	DNA damage repair	^76^
	SP-1	Cys68 and Cys755	Maintain the stability of metabolites and protect endothelial cells	^78^
		Cys664	Inhibit cardiac hypertrophy	^79^
	IGF1R	Unknown	Inhibit the proliferation of vascular smooth muscle cells	^80^
	Kir6.1	Cys43	Vasodilatation	^81^
Nervous system	Parkin	Unknown	Neuroprotective effect	^87^^,^^88^
	CTSS	Cys25	Inhibit ATP-induced neuroinflammation and Aβ1–42 synthesis	^97^
	GSK3β	Cys218	Neuroprotective effect	^98^
	GAPDH	Cys150	Cognitive dysfunction	^106^
	SR	Unknown	Improve synaptic function	^113^
	PKA, PKC, CAMKII	Unknown	Mediate rapid excitatory synaptic transmission	^117^
Kidney	Keap-1	Cys151	Promote cells from oxidative stress	^118^^,^^119^
	GAPDH	Cys150	Increase ATP production	^121^
		Cys156 or Cys152	Decrease ATP production	^115^^,^^120^
	EGFR	C797/C798	Urination and excrete sodium, and lower blood pressure	^123^
	Sirt1	Unknown	Regulation epigenetic function	^125^
Liver	PGC-1α, Glucose-6-phosphatase, Fructose-1, 6-bisphosphatase	Unknown	Enhance the liver glucose produce	^129^
Testis	PDE	Unknown	Restore testosterone synthesis	^130^
Myoblasts	metallothionein-1	Unknown	Protects myoblasts from oxidative stress induced by cadmium	^131^
Melanoma cells	Human serum albumin	Cys34	Exert anti-oxidative stress, inhibit tyrosinase and melanin accumulation	^132^

CaMKII: Ca^2+^/calmodulin-dependent protein kinase II; CTSS: Cathepsin S; EGFR: endothelial growth factor receptor; ER: endoplasmic reticulum; GSK3β: glycogen synthase kinase 3β; HuR: human antigen R; IGF1R: Insulin-like growth factor-1 receptor; Keap-1: Kelch-like ECH-associated protein1; Kir6.1: An ATP-sensitive potassium channel; MEK1: mitogen-activated extracellular signal-regulated kinase 1; MuRF1: muscle RING finger-1; Parkin: An E3 ubiquitin ligase; PDE: phosphodiesterase; PDI: protein disulphide isomerase; PGC-1α: peroxisome proliferator-activated receptor-γ coactivator-1α; PPARγ: peroxisome proliferator-activated receptor γ, PTP1B: protein tyrosine phosphatase 1B; Sirt1: silent mating-type information regulator 2 homolog 1; SP1: specific protein-1, SR: serine racemase; TRPV4 and TRPV1: transient receptor potential family member.

### S-sulfhydration in the cardiovascular system

The presence of endogenous H_2_S has been detected in the arteries and hearts of many animals, including humans,^48^^,^^49^ and has been proven to be widely involved in physiological and pathological processes, such as vasodilation, arterial contraction, cardioprotection, and plaque formation.^50^, ^51^, ^52^ Peroxisome proliferator-activated receptor γ (PPARγ) is the leading factor of blood glucose and lipid metabolism. H_2_S can directly S-sulfhydrate PPARγ at the Cys139 site, increasing nuclear accumulation of PPARγ. It enhanced the DNA binding activity of PPARγ response element promoter and promoted the expression of adipogenic gene, and the mutation of PPARγ at Cys139 site blocked the S-sulfhydration of H_2_S.^53^ Protein tyrosine phosphatase 1B (PTP1B) plays an important role in endoplasmic reticulum (ER) stress and is thought to play an important role in obesity-induced cardiomyopathy and septic shock-induced cardiovascular dysfunction.^54^^,^^55^ H_2_S induces inhibition of PTP1B activity by S-sulfhydration at Cys215, promoting phosphorylation and activation of protease-like ER kinases, and restoring ER homeostasis, all of which are absent in CSE knockout HeLa cells.^56^ Muscle RING finger-1 (MuRF1) is a ubiquitin-linked enzyme, which was first found to play a role in skeletal muscle atrophy and cardiomyopathy.^57^ It is specifically expressed in the M-line and cytoplasm of cardiomyocytes and can degrade sarcomere including troponin I, troponin T, and titin.^58^ Knockdown MuRF1, mutation of MuRF1 at Cys44 in neonatal rat cardiomyocytes, or S-sulfhydration of MuRF1 by NaHS at Cys44 can reduce the protection of diabetic patients from MuRF1 induced degradation of cardiac structural proteins.^59^ These results suggest that S-sulfhydration may play an important role in cardiovascular disease induced by abnormal glucose and lipid metabolism.

It has been proposed that H_2_S -induced protein S-sulfhydration may be a new therapeutic target for preventing diabetes from accelerating atherosclerosis.^60^ Kelch-like ECH-associated protein1 (Keap-1) acts as a negative receptor for nuclear factor E2-related factor 2 (Nrf2) to protect cells from oxidative stress. In human gastric epithelial cells, NaHS S-sulfhydrates Keap-1, promotes Keap-1/Nrf2 dissociation, and increases Nrf2 transcriptional activity, thereby reducing ischemia-reperfusion induced oxidative stress and cell damage.^61^ Keap-1 is S-sulfhydrated in embryonic fibroblasts of wild-type (WT) mice, but not in mice with CSE knockdown. NaHS S-sulfhydrates Keap-1 at Cys151 regulates the localization, activity, and target gene expression of Nrf2 in mouse embryonic fibroblasts. The Cys151 mutation weakened the S-sulfhydration of Keap-1, failed to promote the nuclear translocation of Nrf2, and prevented cell senescence. Defects in a functional domain of Keap-1 (Tramtrack and Bric-á-Brac 2 dimerization domain) eliminated NaHS-induced S-sulfhydration. GYY4137 (an H_2_S donor) reduced arteriosclerotic plaque formation and reactive oxygen species (ROS) levels in LDL-receptor knockout (*LDLr*^*−/−*^) mice, but not in *LDLr*^*−/−*^ and *Nrf2*^*−/−*^ knockout mice. Similarly, foam cells and oxidative stress levels in peritoneal macrophages isolated from WT mice were reduced by GYY4137, whereas those isolated from *Nrf2*^*−/−*^ were not.^62^ In endothelial cells stimulated by oxidized LDL and high glucose, the S-sulfhydration of GYY4137 on Keap-1 at Cys151 and Cys273 promotes the dissociation of Keap-1/Nrf2, while mutations at Cys151A eliminate GYY4137-induced Keap-1/Nrf2 dissociation, Nrf2 nuclear translocation, and ROS clearance.^60^ Protein disulphide isomerase (PDI) activity is closely related to endothelial function and is up-regulated in plaques. ROS manages cytoplasmic PDI activity, ROS-mediated specific oxidase regulates ER PDI activity, and H_2_S has an anti-oxidative stress effect and can directly remove hyperhomocysteinaemia (HHcy) induced ROS.^63^^,^^64^ S-sulfhydration of PDI by NaHS and GYY4137 at Cys53/Cys57 and Cys397/Cys400 inhibits HHcy-induced PDI dysfunction and ER stress, and reduces atherosclerotic plaque formation.^65^ Both CD62E and cathepsin S (CTSS) are associated with endothelial cell activation and atherosclerosis. The constitutive S-sulfhydration of CSE-derived H_2_S at Cys13 reduces the dimer and activity of human antigen R (HuR), thereby preventing its binding to target mRNA and reducing the expression of target protein (for example CD62E and CTSS). However, due to vascular inflammation, Ser377 of CSE is phosphorylated, its activity is inhibited, and CD62E expression is increased, accelerating the formation of endothelial dysfunction and atherosclerosis. Similarly, atherosclerotic plaques increased and lumen area decreased in carotid arteries in mice with CSE knockdown, this can be reversed by SG1002 (a slow-releasing polysulfide donor).^66^

H_2_S regulates voltage-activated calcium channels in the cardiovascular system.^67^^,^^68^ The concentration of NaHS dependently inhibits L-type calcium currents in cardiomyocytes, which can be eliminated by DTT (dithiothreitol, a small molecule organic reducing agent), and NaHS reduces functional free sulfhydryl groups in L-type calcium channels, providing indirect evidence for H_2_S-mediated S-sulfhydration in voltage-activated Ca^2+^ channels.^69^ Transient receptor potential (TRP) channels are Ca^2+^ osmotic ion channels with angiogenic effect and are also regulated by H_2_S.^70^ Inhibition of TRPV4 (TRP family member) inhibited the vasodilation of H_2_S-induced Ca^2+^ and K^+^ influx, while Na_2_S treatment of aortic endothelial cells enhanced the S-sulfhydration of TRPV4. H_2_S-mediated vasodilation required the activation of TRPV4-dependent Ca^2+^ influx in endothelial cells. In addition, TRPV4 causes vasodilation through Ca^2+^ and is enhanced by S-sulfhydration.^71^ In the carotid sinus of spontaneously hypertensive rats, endogenous H_2_S and CBS regulate blood pressure through TRPV1 (another TRP family member), and NaHS supplementation increases the expression of mRNA, protein, and S-sulfhydrated TRPV1 to enhance the sensitivity of carotid baroreceptors and thus dilate blood vessels.^72^ Ca^2+^/calmodulin-dependent protein kinase II (CaMKII) mediates the mitochondrial damage in patients with heart failure by regulating cardiac excitation-contraction coupling, apoptosis, Ca^2+^ homeostasis, and ROS metabolism.^73^^,^^74^ CaMKII activity affects cardiomyocyte Ca^2+^ homeostasis, intracellular Ca^2+^ flux, and mitochondrial Ca^2+^ processing. The S-sulfhydration of CaMKII by H_2_S at Cys6 inhibits the activity of CaMKII, thus improving mitochondrial function, inhibiting mitochondrial apoptosis, and protecting myocardial cells.^75^

**Figure 2:**
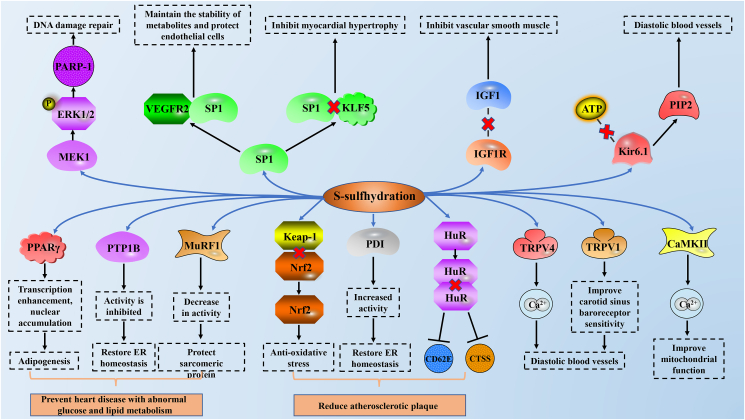
The role of different proteins in the cardiovascular system after S-sulfhydration. After protein S-sulfhydration, it exerts anti-oxidative stress in the cardiovascular system, restores ER homeostasis, regulates ion channels, and regulates glucose and lipid metabolism. It plays a role in blood pressure regulation, atherosclerotic plaque formation, and cardiac hypertrophy. CaMKII: Ca^2+^/calmodulin-dependent protein kinase II; CTSS: cathepsin S; ER: endoplasmic reticulum; ERK1/2: extracellular regulated protein kinase 1/2; HuR: human antigen R; IGF1: insulin-like growth factor-1; IGF1R: insulin-like growth factor-1 receptor; Keap-1: Kelch-like ECH-associated protein1; Kir6.1: ATP-sensitive potassium channel 6.1; KLF5: Krüppel-like factor 5; MEK1: mitogen-activated extracellular signal-regulated kinase 1; MuRF1: muscle RING finger-1; Nrf2: nuclear factor E2-related factor 2; PARP-1: poly (ADP-ribose) polymerase-1; PDI: protein disulphide isomerase; PIP2: phosphatidylinositol (4,5)-bisphosphate; PPARγ：peroxisome proliferator-activated receptor γ; PTP1B: protein tyrosine phosphatase 1B; SP1: specific protein-1; TRPV4 and TRPV1: transient receptor potential family member; VEGFR2: vascular endothelial growth factor receptor 2.

Moreover, in human endothelial cells and fibroblasts, H_2_S induces S-sulfhydration of mitogen-activated extracellular signal-regulated kinase 1 (MEK1) at Cys341 to promote phosphorylation and nuclear transfer of extracellular regulated protein kinases (ERK1/2), thereby activating poly (ADP-ribose) polymerase-1 (PARP-1) and mediating DNA damage repair.^76^ Specific protein-1 (SP-1) is an important transcription factor with multiple functions in the cardiovascular system.^77^ The S-sulfhydration of SP-1 by NaHS at Cys68 and Cys755 can stabilize and enhance the binding of SP-1 to the vascular endothelial growth factor receptor 2 promoter, maintain the stability of metabolites and protect endothelial cells.^78^ H_2_S reduces the promoter activity of Krüppel-like factor 5 (KLF5) and the expression of mRNA, and the S-sulfhydration of SP-1 by H_2_S at Cys664 weaken the activity of SP-1 and KLF5 promoter binding and inhibits cardiac hypertrophy.^79^ H_2_S inhibits the expression of insulin-like growth factor-1 receptor (IGF1R), and the binding ability of S-sulfhydrated IGF1R and IGF1 is weakened, thereby inhibiting the proliferation of vascular smooth muscle cells.^80^ The S-sulfhydration of H_2_S to Kir6.1 (an ATP-sensitive potassium channel) at Cys43 reduces the binding of Kir6.1-ATP and enhances the binding of Kir6.1-phosphatidylinositol (4,5)-bisphosphate to activate Kir6.1, increase the activity of K_ATP_ channels and relax blood vessels.^81^ All of these S-sulfhydrations play an important role in the physiological and pathological processes of the cardiovascular system, but the interaction between them needs to be further explored (Fig. 2).

### S-sulfhydration in the nervous system

The study of H_2_S in the central nervous system began with the discovery of sulfide in the brain, and then the synthesis of endogenous H_2_S was detected in the brain.^82^^,^^83^ Importantly, the metabolic disorder of H_2_S is involved in the development of various neurological diseases. Such as Alzheimer's disease (AD), Parkinson's disease (PD), Huntington's disease (HD).^84^ Parkin, as an E3 ubiquitin ligase, can ubiquitinate the target protein of the proteasome. The mutation of parkin leads to the death of dopaminergic cells in the development of PD.^85^^,^^86^ The S-sulfhydration of parkin is related to its activity, and the S-nitrosylation weakens its activity. Studies have shown that the parkin of S-sulfhydration in PD patients is significantly reduced, which means that the activity of Parkin is closely related to PD.^87^^,^^88^ The S-sulfhydration of H_2_S on parkin enhances its activity, thereby exerting a neuroprotective effect in PD. AD is a progressive neurodegenerative disease with insidious onset. Clinically, it is characterized by general dementia such as memory impairment, aphasia, apraxia, agnosia, impairment of visual-spatial skills, executive dysfunction, and personality and behavior changes.^89^^,^^90^ Although the cause of AD is unknown so far, H_2_S is believed to be involved in the development of AD.^91^^,^^92^ Neuroinflammation and excessive Aβ deposition synergistically promote the development of AD,^93^ signal transducer and activator of transcription 3 (STAT3) mediates neuroinflammation and Aβ deposition in the process of AD, and the inhibition of CTSS in microglia can produce neuroprotective effects in AD.^94^, ^95^, ^96^ In mouse glial cells BV2, H_2_S reduces ATP-induced ROS production, inflammatory response, and Aβ_1-42_ deposition by eliminating phosphorylation of STAT3 and S-sulfhydration of CTSS at Cys25.^97^ The S-sulfhydration of glycogen synthase kinase 3β (GSK3β) by H_2_S at Ctys218 inhibits the phosphorylation of Tau (a major component of the neurofibrillary tangles) beyond Ser396, while the hyperphosphorylation of Tau reduces its affinity for microtubules and causes its aggregation.^98^ In addition, it was found in the autopsy of AD patients and mice that the expression of CSE and the effect of S-sulfhydration decreased, indicating that the increase in the content of H_2_S and the degree of S-sulfhydration can inhibit the progression of AD.^98^^,^^99^

**Figure 3:**
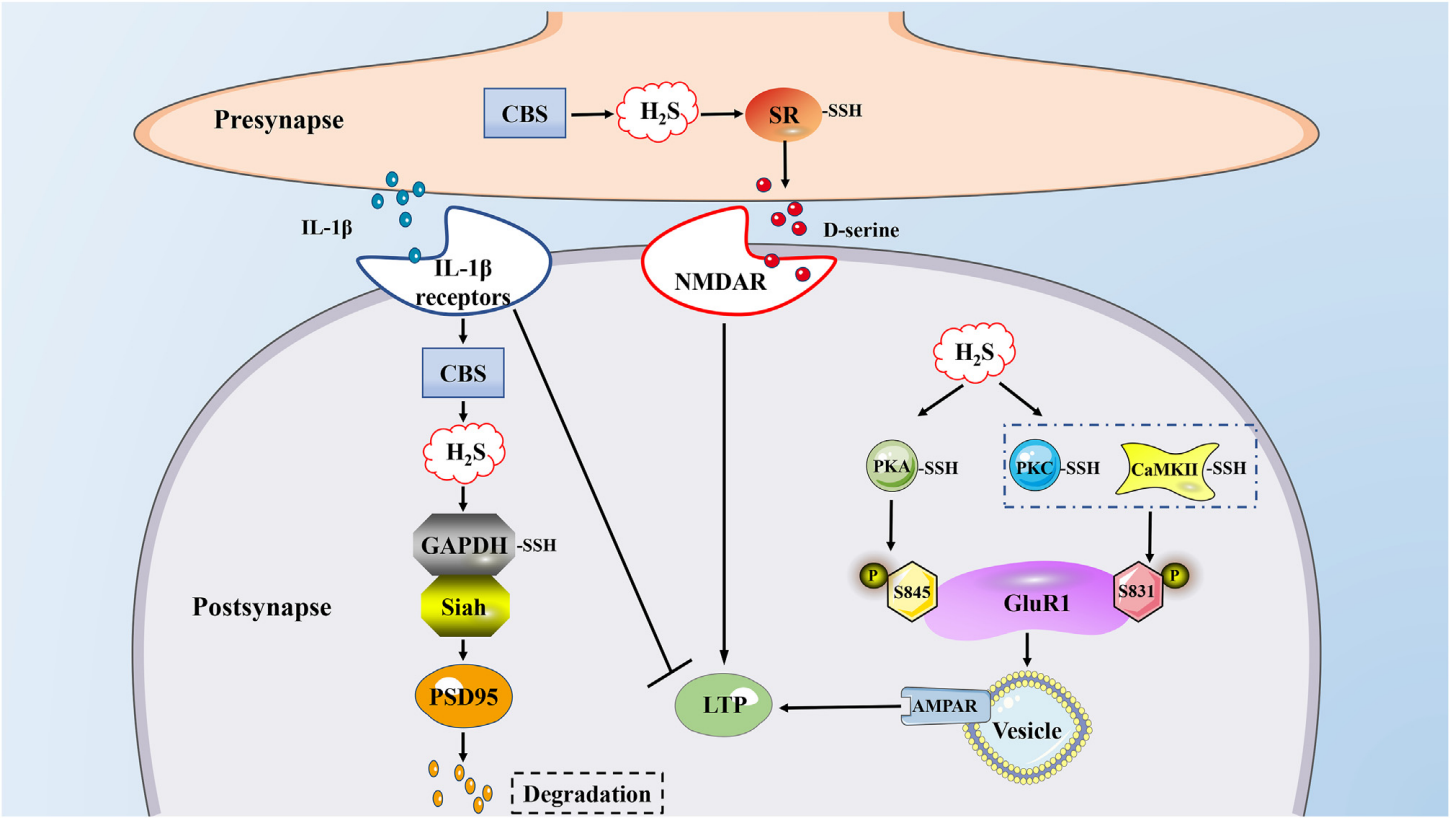
S-sulfhydration plays a role in synapses and participates in memory formation. H_2_S participates in memory formation through S-sulfhydration of GAPDH, SR, PKA, PKC, and CaMKII, which may all be related to LTP. AMPAR: α-amino-3-hydroxy-5-methyl-4-isoxazolepropionic acid receptor; CaMKII: Ca^2+^/calmodulin-dependent protein kinase II; CBS: cystathionine β-synthase; GluR1: AMPAR subunit; LTP: long-term potentiation; NMDAR: N-methyl-d-aspartate subtype glutamate receptor; PSD95: postsynaptic density 95; Siah: E3 ubiquitin protein ligase; SR: serine racemase.

Memory impairment exists in several neurodegenerative diseases and is caused by a variety of pathological and physiological mechanisms, including neuroinflammation and aging.^100^, ^101^, ^102^ Interleukin-1β (IL-1β) is a pro-inflammatory factor widely found in the brain,^103^ postsynaptic density 95 (PSD95) is the core scaffold protein of the postsynaptic protein network, which is involved in the regulation of synaptic stability, strength, and plasticity.^104^^,^^105^ IL-1β plays a crucial role in learning and memory by regulating PSD95. IL-1β mediates the increase of H_2_S and leads to the decrease of neuronal PSD95. This effect is due to the S-sulfhydration of glyceraldehyde 3-phosphate dehydrogenase (GAPDH) at Cys150 by H_2_S, which enhances the binding of GAPDH and Siah (an E3 ligase protein), and enhances IL-1β-induced ubiquitination mediation. The induced PSD95 degrades, which in turn leads to cognitive dysfunction. When CBS is knockout, IL-1β-induced synapse reduction and memory impairment are significantly alleviated. This study provides a new explanation for inflammation-induced memory impairment: IL-1β through the S-sulfhydration of GAPDH by H_2_S leads to the combination of GAPDH and Siah and increases the stability of Siah. Stable Siah promotes the ubiquitination of PSD95.^106^ Long-term potentiation (LTP), a cellular model for memory, is bidirectionally regulated by various redox signals.^107^^,^^108^ The increase of LTP is closely related to the improvement of synaptic function related to aging.^109^^,^^110^ Studies have shown that LTP damage in elderly animals is related to the decrease in N-methyl-d-aspartate subtype glutamate receptor (NMDAR) activity induced by endogenous d-serine.^111^^,^^112^ H_2_S increases the availability of d-serine through the S-sulfhydration of serine racemase (SR) to enhance LTP, and the increase of SR S-sulfhydration reduces the S-nitrosylated SR and enhances the activity of SR. CBS knockout and drug inhibition both damage hippocampal LTP, and this effect can be reversed by supplementation of exogenous H_2_S and d-serine. In addition, after knocking out the SR in the hippocampus, the effect of H_2_S on SR was eliminated, indicating that H_2_S regulates d-serine through S-sulfhydrates SR and plays an important role in hippocampal LTP. In summary, this study shows that S-sulfhydration of SR improves NMDAR-dependent synaptic function. There are pieces of literature that LTP and memory in the brain are also related to the concentration of IL-1β, that is, high concentrations of IL-1β inhibit synaptic strength and LTP,^113^ while physiological levels of IL-1β promote LTP and the formation of memory,^114^ and the increase in IL-1β causes an increase in the production of H_2_S.^106^ Therefore, whether the concentration of IL-1β affects LTP remains to be verified. In addition, GAPDH, a key regulator of astrocyte SR, is S-sulfhydrated in the kidney and synapse, but its effect on the astrocyte needs to be further clarified.^106^^,^^115^ α-Amino-3-hydroxy-5-methyl-4-isoxazolepropionic acid receptor (AMPAR) mediates rapid excitatory synaptic transmission in the central nervous system, and its dynamic expression in the postsynaptic membrane is related to the induction and maintenance of LTP and long-term depression (LTD), and participates in the regulation of learning and memory activities. Phosphorylation of AMPAR subunits in neurons is an important regulatory mechanism that controls their functions.^116^ A variety of protein kinases (such as PKA, PKC, CAMKII) and protein phosphatase type 2A are key regulators of AMPAR phosphorylation. H_2_S can enhance the activity of PKA, PKC, and CAMKII through S-sulfhydration, thereby phosphorylating GluR1 (AMPAR subunit) at Ser845 and Ser841, consequently increasing the surface activity of GluR1, and these effects can be reversed by DTT.^117^ To sum up, H_2_S affects the formation of memory in many ways in the nervous system, and the generation of H_2_S in different parts may have the opposite effect. This may be related to the bell-shaped pharmacological model of H_2_S.^13^ In short, H_2_S impacts on memory needs further study (Fig. 3).

### S-sulfhydration in kidney

**Figure 4:**
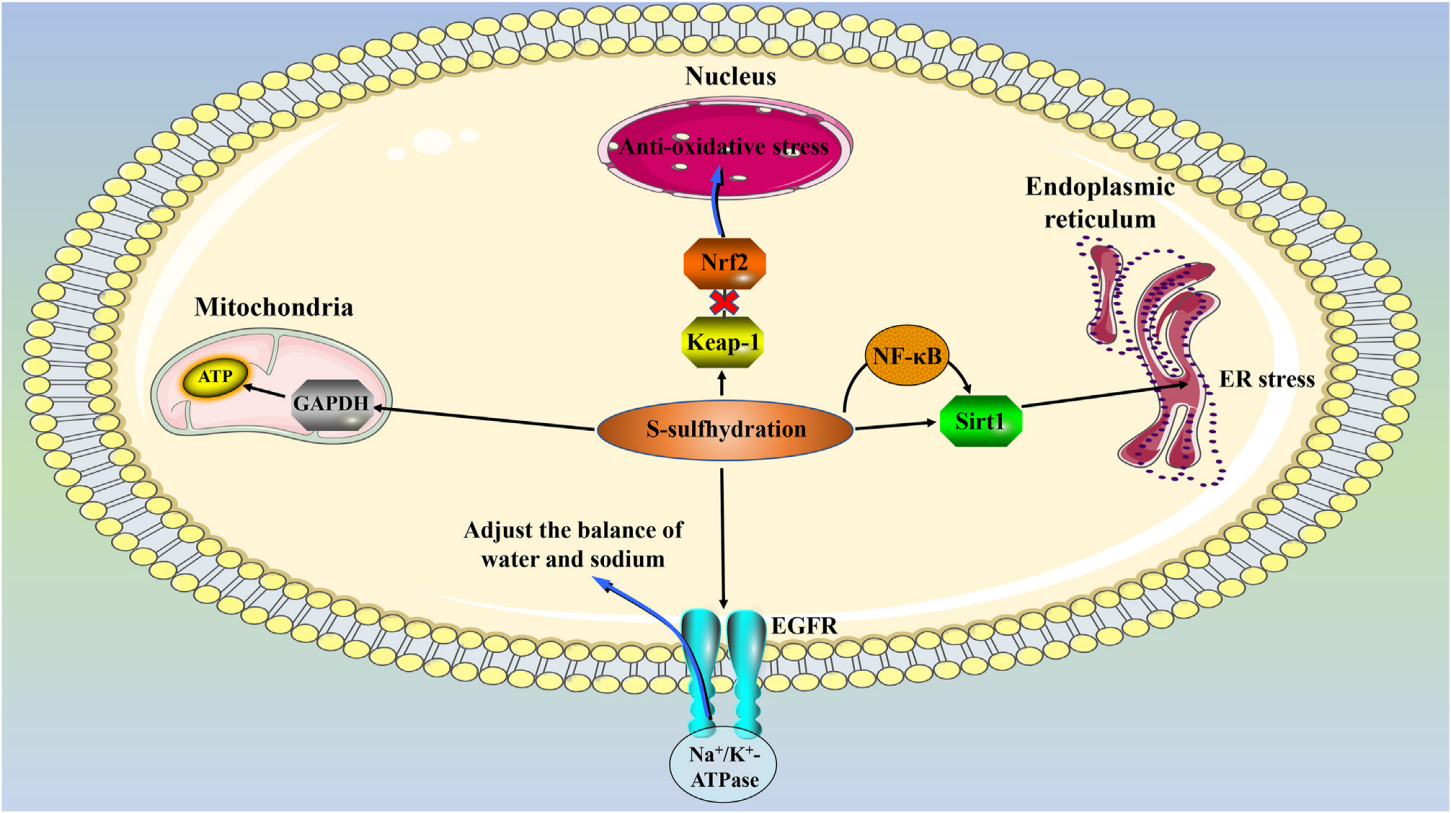
The role of S-sulfhydration in the kidney. S-sulfhydration regulates ATP production, anti-oxidative stress, regulation of ER stress and water-sodium balance in the kidney. EGFR: endothelial growth factor receptor; ER: endoplasmic reticulum; Keap-1: Kelch-like ECH-associated protein1; Nrf2: nuclear factor E2-related factor 2; Sirt1: silent mating-type information regulator 2 homolog 1.

H_2_S plays an important role in the physiological and pathological processes of the kidneys by regulating the blood flow, endocrine, and metabolic functions of the kidneys. Its S-sulfhydration to proteins acts on the kidneys mainly through the following channels: 1) Keap-1, which plays a role in the cardiovascular system, also plays a role in the kidney. The S-sulfhydration of Keap-1 at Cys151 by H_2_S dissociates the transcription factor Nrf-2, promotes the transfer of Nrf-2 to the nucleus, and promotes cells from oxidative stress.^60^ And Nrf-2 is thought to play a role in artificially induced kidney damage after ischemia-reperfusion.^118^^,^^119^ 2) The S-sulfhydration of GAPDH not only affects its activity but also protects GAPDH from harmful PTM. The S-sulfhydration of GAPDH at Cys150 enhances its activity, and the S-sulfhydration at Cys156 or Cys152 weakens its activity.^115^^,^^120^ At the same time, the S-sulfhydration of Cys150 also competitively weakens the S-nitrosylation at Cys150, and the S-nitrosylation will weaken the activity of GAPDH.^121^ GAPDH activity is positively correlated with ATP production. In acute kidney injury, the production of ATP is essential for the recovery of renal function.^122^ 3) S-sulfhydration of proteins can also adjust the balance of water and sodium. S-sulfhydration at the C797/C798 site of endothelial growth factor receptor (EGFR) induces the endocytosis of Na^+^/K^+^-ATPase, which leads to the loss of renal tubular Na^+^/K^+^-ATPase function, induces mice to urination and excrete sodium, and lower blood pressure.^123^ 4) Silent mating-type information regulator 2 homolog 1 (Sirt1) participates in aging, metabolism, and tolerance to oxidative stress by deacetylating its various substrates (including histones, transcription factors, and co-regulators).^124^ H_2_S can not only directly S-sulfhydrate Sirt1 to increase its activity, but also increase the expression of Sirt1 through S-sulfhydration of its upstream transcription factor NF-κβ, and the S-sulfhydration of Sirt1 also reduces its deacetylation ability, which plays an important role in the regulation of its epigenetic function.^125^, ^126^, ^127^ In addition, H_2_S also S-sulfhydrates certain proteins in the PI3K/Akt, ERK1/2, and ATF4 pathways, thereby reducing ER stress^76^^,^^128^ (Fig. 4).

### S-sulfhydration in other systems

In addition, in hepatocytes, H_2_S can regulate the expression of peroxisome proliferator-activated receptor-γ coactivator-1α (PGC-1α) through glucocorticoid receptor and cAMP/PKA pathways, can also directly S-sulfhydrate PGC-1a to enhance its activity, and can also S-sulfhydrate glucose-6-phosphatase and fructose-1,6-bisphosphatase to enhance the liver glucose produce.^129^ In the testis, CBS-catalyzed H_2_S inhibits phosphodiesterase (PDE) expression through S-sulfhydrated PDE, activates the cAMP/PKA pathway, and restores testosterone synthesis.^130^ In myoblasts, CSE-induced H_2_S protects myoblasts from oxidative stress induced by cadmium by S-sulfhydrating metallothionein-1.^131^ In melanoma cells, H_2_S S-sulfhydrates human serum albumin at Cys34 to exert anti-oxidative stress and inhibit tyrosinase and melanin accumulation.^132^

## S-sulfhydration and other PTMs

Except for S-sulfhydration, no other PTMs directly induced by H_2_S have been found for the time being. But proteins S-sulfhydration can indirectly regulate other types of PTM, such as inducing protein phosphorylation, competitive inhibition of S-nitrosylation, methylation, etc.^56^^,^^121^ It is unclear whether H_2_S can induce other types of PTM to produce biological effects.

## The detection method and principle of S-sulfhydration

The current methods for detecting S-sulfhydration mainly include modified biotin-switch technique, N-ethyl maleimide blocking method, tag-switch assay. The modified biotin-switch technique utilizes the property of the thiol blocking agent iodoacetic acid (IAA) to react with free thiols and protein persulfides. IAA reacts with S-sulfhydrated proteins to form alkylated S-sulfhydrated compounds, which are then cleaved with dithiothreitol (DTT) to modify the compounds. Specific cysteine was then labeled with iodoacetamide-linked biotin. However, in this method, DTT will also cleave S-nitrosothiols, resulting in errors.^56^ Maleimide selectively interacts with the sulfhydryl group of cysteine and labels sulfhydrated and non-sulfhydrated cysteines. The use of fluorescently labeled maleimide forms adducted disulfides with persulfides in the sample, which are decomposed by DTT resulting in a decrease in fluorescence. Because of the availability of commercial reagents, this method is relatively simple and can be quantitatively analyzed. This method cannot be used to label S-sulfhydrated proteins because persulfides are already involved in the reaction.^127^ The tag-switch assay uses two reagents to label protein persulfides in two steps. The first step uses a thiol blocker (methylsulfonyl benzothiazole) to label-free thiols and persulfides. When properly labeled, disulfide bonds in persulfide adducts can exhibit higher reactivity with certain nucleophiles than those commonly found in proteins. Therefore, only persulfide adducts can be labeled with available nucleophile-containing tag-switching reagent. In the second step, the persulfide adduct can be labeled with a labeled cyanoacetic acid derivative (nucleophile). The advantage of this method is that the intracellular S-sulfhydration can be observed under a fluorescence microscope.

These methods have their advantages and disadvantages,^133^, ^134^, ^135^ therefore, more effective methods need to be developed to identify the effects and sites of S-sulfhydration.

## H_2_S donors and their possible challenges as a drug

Since the discovery of the physiological function of H_2_S, more and more H_2_S donors have also been discovered or synthesized. Among the natural compounds, there are not only inorganic substances such as NaHS, Na_2_S, but also Allicin, Ajoene, Diallyl sulfide, Diallyl disulfide from garlic and sulforaphane from cruciferous plants. The anti-inflammatory and anticancer effects of garlic and cruciferous plants may be related to H_2_S donors.^136^, ^137^, ^138^, ^139^, ^140^, ^141^, ^142^, ^143^ Synthetic compounds such as GYY4137, 5-(4-hydroxyphenyl)-3H-1,2-dithiole-3-thione, S-propargyl-cysteine are pure H_2_S donors.^144^^,^^145^ In recent years, the number of compounds developed in conjunction with other drugs or donors has gradually increased, such as the non-steroidal anti-inflammatory drug H_2_S-NSAIDs containing H_2_S,^146^ and there are NOSH-aspirin and NOSH-sulindac containing H_2_S and NO donors developed using the synergy of NO and H_2_S.^147^^,^^148^

Since the concentration of H_2_S in living cells is difficult to measure, the concentration of the donor is now used as the drug concentration, but high concentrations of H_2_S are toxic to the organism, and different tissues have different tolerance to H_2_S. Therefore, how to target H_2_S donors to target tissues and maintain appropriate concentrations is a challenge for drug development. Even so, individual differences are also issues that need to be considered.

## Concluding remarks and prospects

The cellular and biological effects of H_2_S are mainly mediated through the following ways: (1) interaction with ion channels, (2) mutual effect with second messengers, (3) direct or indirect PTM, (4) regulation Redox balance, and (5) oxygen sensing and mitochondrial bioenergetics.^149^, ^150^, ^151^ However, as the research progresses, S-sulfhydration, this kind of PTM directly induced by H_2_S, has attracted more and more attention. S-sulfhydration has been shown to be involved in all the biological effects of H_2_S.

Protein S-sulfhydration is a post-translational modification directly induced by H_2_S, which may be the molecular mechanism of H_2_S action. However, not all S-sulfhydration can change the spatial structure and activity of a protein. Even the S-sulfhydration of the same protein can exhibit different effects, which may be determined by the position of S-sulfhydration cysteine residues. If the S-sulfhydrated cysteine locates in a key structural domain, it is essential to maintain the activity and function of the protein. However, the exact nature of the crosstalk between S-sulfhydration and other PTMs is still unclear and needs to be further elucidated.

Although there are many problems to be solved, the importance of S-sulfhydration is self-evident. More information about S-sulfhydration will help us understand the role of S-sulfhydration in the mechanism of H_2_S. In addition, protein S-sulfhydration (induction or blocking) may become a potential new target for drug design, this may provide a new direction for the development and application of H_2_S-based drugs.

## Author contributions

DW, HC, and XJ conceived the study and drafted the manuscript. HC, LQ, KL, YQ, and JZ prepared the figures. All authors read and approved the final manuscript.

## Conflict of interests

The authors declare that they have no competing interests.

## Funding

This work was supported by grants from the 10.13039/501100001809National Natural Science Foundation of China (No. 81802718 and 81670088), the Training Program for Young Backbone Teachers of Institutions of Higher Learning in Henan Province, China (No. 2020GGJS038), and the Foundation of Science & Technology Department of Henan Province, China (No. 202102310480, 222102310490, and 222102310495).
